# Examining Human Unipedal Quiet Stance: Characterizing Control through Jerk

**DOI:** 10.1155/2020/5658321

**Published:** 2020-01-04

**Authors:** Matthew R. Semak, Jeremiah Schwartz, Gary Heise

**Affiliations:** ^1^Department of Physics and Astronomy, University of Northern Colorado, Greeley, CO 80639, USA; ^2^School of Sport and Exercise Science, University of Northern Colorado, Greeley, CO 80639, USA

## Abstract

We investigated the quality of smoothness during human unipedal quiet stance. Smoothness is quantified by the time rate of change of the accelerations, or jerks, associated with the motion of the foot and can be seen as an indicative of how controlled the balance process is. To become more acquainted with this as a quantity, we wanted to establish whether or not it can be modeled as a (stationary) stochastic process and, if so, explore its temporal scaling behavior. Specifically, our study focused on the jerk concerning the center-of-pressure (COP) for each foot. Data were collected via a force plate for individuals attempting to maintain upright posture using one leg (with eyes open). Positive tests for stochasticity allowed us to treat the time series as a stochastic process and, given this, we took the jerk to be proportional to the increment of the force realizations. Detrended fluctuation analysis was the primary tool used to explore the scaling behavior. Results suggest that both the medial-lateral and anterior-posterior components of the jerk display persistent and antipersistent correlations which can be modeled by fractional Gaussian noise over three different temporal scaling regions. Finally, we discussed certain possible implications of these features such as a jerk-based control over the force on the foot's COP.

## 1. Introduction

The analysis of human postural control has become more complex in the past several decades. Still, much of these efforts study the simple motion of the center of pressure (COP) which is the point of application of a person's net force on the surface supporting the body. Using a force plate for this surface, one can record force information as a function of time for a balance session and study, for example, the COP's trajectory, range of motion, and velocity [1]. The seminal work of Collins and De Luca [2] introduced an approach to investigate bipedal quiet stance by studying stabilograms (a two-dimensional plot of the COP's trajectory) in the context of fractional Brownian motion. They suggested that neuromuscular control can be gleaned from the temporal correlations in the COP trajectory; over short time scales, the COP behaved as a positively correlated random walk, and over long time scales, the COP behaved as a negatively correlated random walk. These correspond to open-loop control and closed-loop control, respectively. Later, Delignieres et al. [3] clarified issues related to the use of fractal-based techniques by Collins and DeLuca and presented an argument as to why postural control is actually velocity-based rather than position-based [4]. Other methodological approaches have examined the Lyapunov exponents of the trajectory of the (sitting) COP [5], evaluated the COP trajectory's information production rate with sample entropy [6, 7], and investigated the fractal character of the temporal evolution of the COP [4, 8, 9]. Recently, an enhanced version of detrended fluctuation analysis with scaling examined for certain frequency components was used to suggest that, at least two time scales, long (position based) and short (velocity based), are involved with postural control [10].

One-legged stance represents a greater challenge to humans and thus allows researchers and clinicians to evaluate the effects of aging health, injury, fatigue, and the influence of visual information [9, 11–21]. In particular, Parreira et al. [11] found significant differences in the changes in COP movement over certain time intervals between young adults and elderly subjects. More specifically, the changes in the area of the COP (Δ A-COP) were calculated and compared for the two age ranges. Δ A-COP increased with increasing time intervals for both groups; however, larger Δ A-COP values were reported for the elderly subjects. The authors concluded that there is a time dependence concerning age-related differences in Δ A-COP. In another example, Jonsson et al. [22] assessed the temporal characteristics of the ground reaction force as healthy young and elderly adults transitioned to one-legged stance from two-legged stance. Two temporal phases were identified in their work: a “dynamic phase” during which the force variability decreased quickly followed by a “static phase” over which the variability remained at a certain level. These authors found that, over the first five seconds of balance, the variability decreased significantly more for the young adults than the elderly. This was seen to result in lower force variability for the young subjects for the subsequent phase.

In this paper, we studied unipedal quiet stance as it should offer structured frequency content beyond that of quiet (two-leg) stance in that the balance control “mechanism,” as mentioned above, is more challenged. Our purpose, unlike many past studies, is to examine the characteristics of the jerk (the time-rate-of-change of the acceleration) associated with the motion of the COP. We would like to use it in gauging the extent to which the balance process is controlled, or “smooth.” This is not so dissimilar from examining the force variability as in [22]. However, we wanted a more detailed view of the temporal changes in the force to, we feel, better address the issue of “smoothness.”

As one can gather, characterizing the process of balance in terms of relevant time scales is of interest as an understanding of the COP's motion is sought. Knowing the temporal scaling/correlations present and the dynamical character of the motion may inform one as to what physiological factors could be at play when a particular control strategy is being employed. However, before looking at the scaling properties, we wanted to characterize the data as to its stationary and stochastic qualities to make a general dynamical statement concerning the jerk. Moreover, our preliminary work appeared to indicate that the force evolves as a nonstationary (certain statistical characteristics change with time [23, 24]) stochastic process. Yet, the increment of the force (which is directly proportional to the jerk) did appear to be a stationary process. If this was so, then we could use the analytical tools available for the study of stationary time series which are lacking for the nonstationary case [23, 25, 26]. In addition, we wanted to make general statements about the stochastic quality of our data for two main reasons. First, we wanted to be able to establish whether the temporal evolution of the jerk (our measure of postural control), while maintaining one-legged balance, should be modeled by a stochastic process. Such a question has been raised in the literature [27]. Second, we needed to be able to interpret any scaling measures in the proper context. For example, we wanted to be able to distinguish between chaotic (deterministic) fractal noise behavior [26].

Next, the scaling behavior was addressed. A rather “coarse-grained” approach was first used that took a root-mean-square of force differences over a range of time scales. This allowed us to examine where scaling regions generally appear as well as quantify the scaling features in a relatively simple way. Then, we looked for (long-range) temporal correlations by performing detrended fluctuation analyses [28] on the time series for the force increments. With this method, we could estimate the memory, if present, and quantify its form via a scaling exponent [29].

Overall, the issue of smoothness is of interest to us as we not only seek a better understanding of variability during the process of human balance but also want to eventually investigate the response of various clinical populations. This is important for assessing sensorimotor issues and motor learning [30]. Moreover, we would like to contribute to the development of a more nuanced control system for robotic applications as well [31].

## 2. The Character of the Data

Force data were collected using a multiaxis force plate (model OR6-6-2000) built by Advanced Mechanical Technology, Inc. This uses a 6-component transducer to measure three force and three moment components. Measurements were taken for five adult subjects who each maintained balance with eyes open looking straight ahead, hands by their sides, and barefoot, using each leg separately for 30 second sessions. The ages of these subjects range from 20 to 60 years (mean being 34 years). Prior to any data collection, all procedures were explained to each participant and written consent was obtained in accordance with the local institutional review board policy.

One foot was placed squarely on the force plate while the other hovered at approximately 30 cm above the plate. These data were sampled at 100 Hz producing a time series for the force generated by medial-lateral (M-L) as well as anterior-posterior (A-P) sways. We found 100 Hz to be an optimal rate as with a collection time of no more than 30 seconds (we were concerned about the effect of fatigue on the subjects), and 3000 data points for each balance session were taken as adequate [32]. Our estimates for certain statistics did not change significantly for data sets with at least 1500 measurements over a 30-second balance session. Moreover, with single-leg balancing, we wanted to be careful to capture any behavior that might need frequencies higher than those in the case of bipedal stance. A Nyquist frequency of 50 Hz may seem high for this study; yet, we would like to be careful in resolving any fractal characteristics which can appear on short time scales [33, 34].

**Figure 1:**
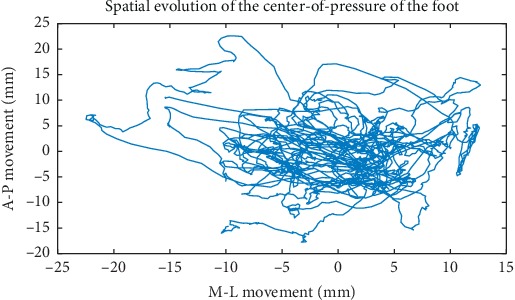
Typical trajectory of the COP for human one-leg stance.

**Figure 2:**
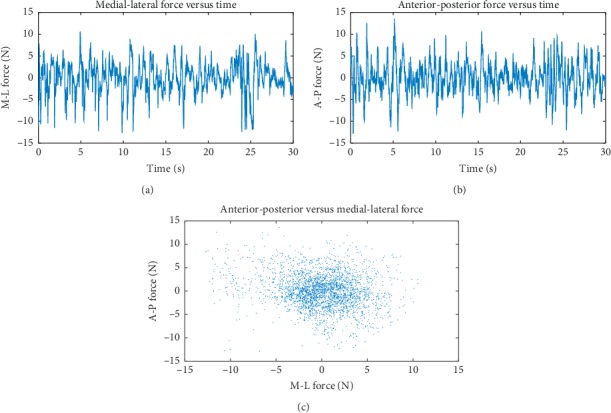
(a, b) These plots portray an example of the temporal evolution of the force during one-leg stance for the M-L and A-P motions. (c) The A-P and M-L forces are plotted against each other. There is no apparent visual correlation between them. Typical time series for (a) the M-L force and (b) the A-P force. (c) A scatter plot of the forces shown in (a) and (b).

A typical COP trajectory for a one-leg balance session is shown in Figure 1. One might note that, in general, it would appear that the area covered by such a trajectory for the case of bipedal quiet stance is less, with greater variance in A-P movement, although there is a similar random character. As with all the data we study, any linear trend in the M-L/A-P movement has been removed. One can appreciate the seemingly complex motion the figure reveals. Figures 2(a) and 2(b) are plots of typical time series for the forces involved in this process.  Figure 2(c) is a plot of A-P versus M-L forces in which no significant correlation is visually obvious. In fact, linear regression estimates resulting in significant models using the M-L force data set as a predictor for A-P force only account for a negligible amount of the variance (less than 5% on average). For this work, we will examine A-P and M-L movement data separately.

**Figure 3:**
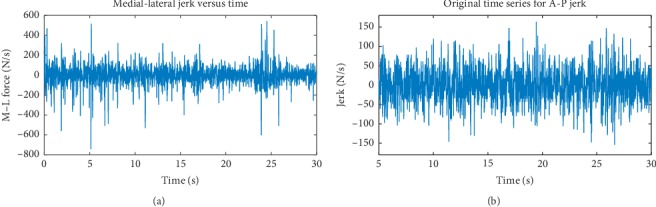
(a, b) Typical examples of the jerk during one-leg stance showing both the M-L and A-P motions. One statistical characteristic of note is that, on average, the standard deviations for the M-L and A-P jerks are 85 N/s and 65 N/s, respectively. Typical time series for (a) the M-L jerkand (b) the A-P jerk.

With the mass of the region of the body under study being constant and our concern ultimately being with variations in the jerk, we did not divide by the mass and took the jerk as simply the time-rate-of-change of the force on the COP. So, we must be careful to state that the reported jerk is scaled by mass. Also, data concerning the force were desirable for our study, not only because it was needed to calculate the jerk, but also it is what our equipment measured directly. Indeed, the position of the foot's center of pressure is derived from force and moment measurements.  Figure 3 shows the jerk corresponding to the plots in Figure 2. Considering the force data as a realization of a stochastic process, the jerk was taken as the increment, Δ*f*=*f*(*i*+1) − *f*(*i*), *i*=1,…, *N* − 1, divided by the sampling period (0.0100 s), where *f*(*i*) is the *i*th realization of the force and *N* is the number of observations making up the time series for the forces.

For all time series considered in this work, any transients were allowed to decay sufficiently before taking estimates as subjects were allowed 5 seconds to stand on the plate before data collection began (checking that the power spectral density did not change significantly). Unlike the work of Jonsson et al. [22] discussed in Introduction, our analysis is limited to “steady state” characteristics so as to be well-defined, given the techniques we are using. Also, we did not filter the frequency range of the data other than having a Nyquist frequency of 50 Hz and linearly detrending. Another important consideration related to temporal evolution was alluded to the above. The quality of stationarity concerns variations in time of the statistics of the time series. We are referring to wide-sense stationarity by which it is meant that the mean and autocovariance of the time series do not change with time [35, 36].

### 2.1. Stationarity Concerns

In an attempt to eliminate the simplest time series features which are not of interest for this study and could lead to nonstationarity, as mentioned above, all times series under study were linearly detrended. This subtracts the best-fit line from the data and allows us to focus on the fluctuations about this line which contains the information of interest for this work. Also, we are concerned with the stationarity of our data in that, as previously mentioned, we would like to use analytic tools (such as a power spectral density/autocorrelation function) which depend on stationarity for them to be well-defined in their application [23]. Also, it should be noted that, to perform a detrended fluctuation analysis (presented below), stationarity is not required [29, 37].

**Figure 4:**
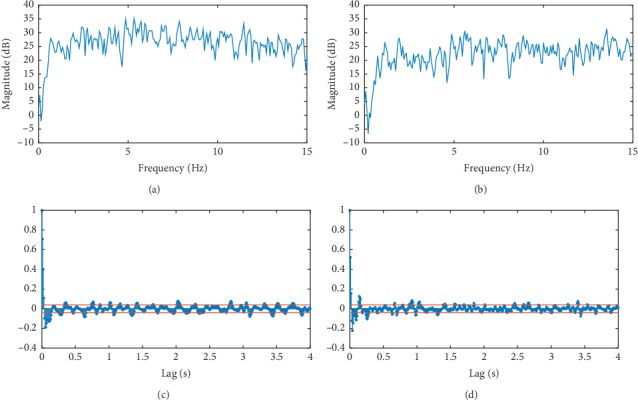
(a, b) The power spectral densities for a subject's M-L and A-P jerk signals. (c, d) Corresponding autocorrelation functions plotted. The horizontal lines indicate 95% confidence bounds. In this case, the relatively rapid falloff of the autocorrelation functions to such small values is indicative of stationary signals. These qualities are generally representative of the jerk data. Power Spectral density of (a) M-L jerk and (b) A-P jerk. (c) Autocorrelation function for (c) M-L jerk and (d) A-P jerk

In Figure 4, one can see the general characteristics typical of the jerk data, given the treatment described above. The power spectral density (estimated using the Welch method [38]) and the autocorrelation function have been estimated for the jerk data [39]. The correlation profiles for jerk with their rapid falloff do appear to be indicative of stationary behavior [35]. For greater lag times, linear correlations, as measured by the autocorrelation function, are subtle at best. This may be indicative of aperiodic oscillations (we will consider this point later in the paper). The time series for the force is rather questionable when it comes to the property of stationarity. Our detrended fluctuation analysis (see below) provided strong evidence against the force having a stationary character [29]. If a process is nonstationary, it is difficult to determine what the autocorrelation function reveals as this function's definition assumes process stationarity [23, 35]. Given this, we feel justified in focusing on the jerk (the main concern of this work) in order to get the most reliable information, given the tools we are using. We, then, did some hypothesis testing in the hope of finding stronger evidence of stationarity.

First, an Augmented Dickey–Fuller test for stationarity was performed on jerk data. Here, again, by stationarity, we mean in the wide- or weak-sense meaning that the mean and autocovariance of the time series do not change with time. This test evaluates the null hypothesis of a unit root existing in a time series sample using a specific autoregressive (AR) model for the process [35, 40–42]. As the name implies, an autoregressive (AR) process is one in which its current value depends on some linear combination of earlier values plus a term taking any new information, or “innovation,” (uncorrelated with the other terms) into account [35]. This common linear model for time series has well-known conditions for stationarity [35, 40, 43]. In particular, if the characteristic polynomial for the AR process has a unit root (the coefficient of the earliest term in the linear combination for the AR process being 1), the process is nonstationary [43]. So, the null hypothesis for this test is that the characteristic equation for the model AR process has a unit root.

Then, a least-squares regression is performed on the jerk data. The form of this regression is dictated by the AR model for the data which requires a certain number of earlier process values to be taken into account (we followed the literature in this regard). Then, the coefficient for the earliest term is tested for. Regarding our data, this test indicates that there is evidence to suggest that these time series are stationary, at least, in the wide-sense. All test critical values were surpassed for each time series with the probability value, *p* < 0.001. This test's statistics also indicated that the data do not have any deterministic trends linear in time that could be removed, i.e., trend stationarity [35] (see the references for a complete description of this hypothesis test).

In addition to the Augmented Dickey–Fuller test, the Phillips–Perron test for stationarity was also used [44]. This is also a unit root test using statistics similar to those used in the Dickey–Fuller test. However, serially correlated errors in the regression process are handled differently. We will let the reader refer to the literature for more information. Overall, all time series were tested and, again, there was significant evidence to suggest that the jerk data are stationary without any deterministic trends linear in time with *p* < 0.001.

Moreover, as suggested in [26], wide-sense stationarity was also tested for by breaking each series into two nonoverlapping pieces and estimating the correlation dimension for each in the limit of small threshold distance. In simple terms, the correlation dimension is a measure of how system values are distributed in phase space [45]. This was done using the usual procedures found in the literature [26, 34, 46, 47]. We found that the dimension for each piece did not vary significantly. (This was true for each of four embedding dimensions.) Then, in addition, we proceeded to perform this test on smaller pieces of each series with the same results. This was not performed as a hypothesis test but as an informal check.

### 2.2. Machine Noise

Machine noise was gauged by examining force data generated for a dormant mass of 20 kg. We chose a moderate mass so as to put the force plate under some load while attempting to still have some indication of the effect of machine idle noise. The M-L and A-P signals for the mass both have standard deviations no higher than 0.2 N (several trials were run for the dormant mass), whereas the average standard deviations for the M-L and A-P signals for the subjects are 4 N and 3 N, respectively.

**Figure 5:**
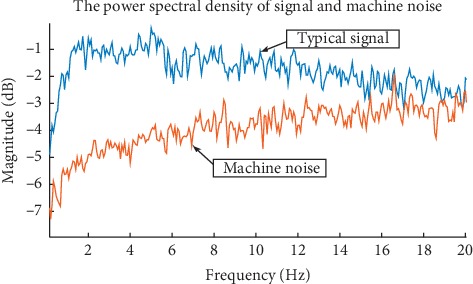
Comparison of the typical signal and machine noise strength using the spectral density. This informed the choice to limit our study to frequencies under 10 Hz.

Given that the stationarity of the jerk signals has been established above and gauging that the machine noise was white for the force and, thus, stationary (along with its increment), a well-defined power spectral density (again, using the Welch method [38]) was generated to compare the strengths of the signal and the noise.  Figure 5 shows a typical situation. The signal strength of the jerk was well distinguished from that of the machine noise for frequencies under 10 Hz. So, we decided to limit the time scales of the study to larger than 0.100 s (under 10 Hz).

### 2.3. Surrogate Data Testing

As mentioned above, we wanted to gain insight into the stochastic and dynamical characters of the time series. For this, we used linear surrogate techniques. For a given set of data, a surrogate data set may be generated. Although different from the original data, the surrogate sets are generated in a way that preserves key properties of the original data. This methodology is appropriate for our stationary data in that periodicity and far reaching trends did not appear to be present (quasiperiodicity could certainly play a role) [24]. We used what are known as Algorithm 0 (testing surrogate time series generated by randomly shuffling the original time series) and Algorithm 1 (testing surrogates constructed using Fourier transforms of the original time series) [24]. We used these two algorithms as for this study we wanted to test primarily the stochastic character of the data to establish the context for properly interpreting any scaling behavior. Any data for which the null hypotheses of Algorithm 0 and 1 can be rejected will be considered questionable at this point (we have pursued testing through Algorithm 2 [24], however with unclear results so far and studies will continue (possibly as in [48])).

We must then choose and calculate a discriminating statistic for the original and surrogate data and, using statistical techniques, see how well these results compare. This will allow us to determine whether or not a null hypothesis can be rejected. Sample entropy, a measure of complexity of the time series, was used for the discriminating statistic [6, 49]. Sample entropy measures the complexity by considering the probability that if subseries, or template vectors, of a certain length *m*, are within a certain distance, or tolerance, then so are template vectors of length *m*+1. We compared template vectors of lengths 3 and 4 data points (median sample entropy was seen to converge at these values for force and jerk data). The unitless tolerance value was varied from 0.20 to 0.60 of the standard deviation of the data set being tested (examining the median maximum relative error, which aids in determining the optimal tolerance value, indicated the comparable error for this tolerance range). For the hypothesis testing, consistent results for this range of tolerances were taken. For a careful discussion of this topic, refer to [6, 7]. A rank-order criterion was used to determine if the null hypothesis for a specific test was to be rejected or not [24]. By this standard, the null hypothesis is to be rejected if the discriminating statistic's value for the original data is higher or lower than any of the statistic's values for the surrogates. To maintain a confidence level of, at least, 95%, 20 surrogates were tested for each original time series.

**Figure 6:**
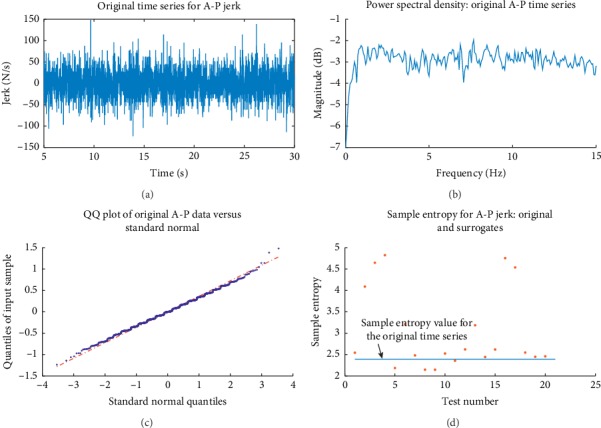
Typical characteristics for data tested using Algorithm 0 with the null hypothesis failing to be rejected. (a) The original A-P time series tested and (b) its corresponding power spectral density. (c) This is a quantile-quantile plot of the original data indicating that these data follow a fairly normal distribution with some asymmetric tail deviation. (d) A rank-ordered criterion was applied for Algorithm 0 (using 21 surrogate data sets) and, as seen here, for this test, there was a failure to reject the null hypothesis. Sample entropy (with a tolerance 0.20 of the standard deviation of the data values) was used in this case. This suggests that the original time series has the character consistent with IID noise. The power spectral density is relatively flat.

Algorithm 0, as mentioned above, was affected by randomly permuting the time series values, thus destroying any linear correlations. Then, the null hypothesis is that there is not a statistically significant difference between the data and uncorrelated (independent and identically distributed (IID)) noise [24]. The null hypothesis failed to be rejected at the 95% level for data sets corresponding to A-P movements for three subjects. These data are suggested to have the character of uncorrelated noise.  Figure 6 shows an example of relevant information rather typical of this situation. The tests for all other data sets showed significant evidence to reject the null hypothesis under Algorithm 0. These data were then tested under Algorithm 1.

**Figure 7:**
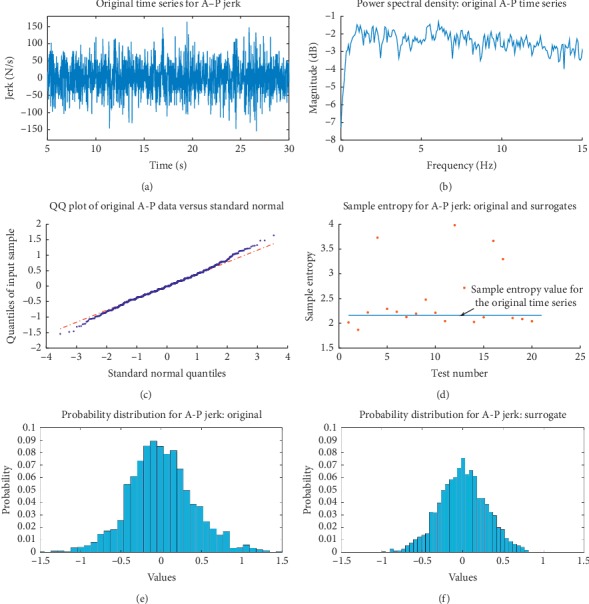
Typical characteristics of data tested using Algorithm 1 with the null hypothesis failing to be rejected. (a) This is an original A-P time series that was tested and (b) its corresponding power spectral density. (c) Quantile-quantile plot of the original data: this indicates that these data follow a fairly normal distribution with a tendency toward fat tails. (d) Again, as with Algorithm 0, a rank-ordered criterion is applied and, here, a failure to reject the null hypothesis is indicated. Sample entropy (with a tolerance 0.20 of the standard deviation of the data values) was used. This corresponds to the original time series having a character consistent with linearly filtered IID noise. (e, f) The power spectral density does show an interesting falloff. The probability distributions for the original and, respectively, one of the surrogates are displayed. They are different as they are expected to use Algorithm 1. However, the difference, at least visually, is not too drastic (as mentioned in the text, this has to do with worries of falsely rejecting the null hypothesis under Algorithm 1, although, in this particular case, it is not a concern [24]).

Under Algorithm 1, the linear correlations of the original data are preserved. This is done through taking a Fourier transform of the original time series and randomizing the phases and leaving the amplitudes unaltered [24]. Surrogates are generated by taking the inverse transform back to the time domain. These surrogates are consistent with the null hypothesis of data being linearly filtered IID noise. This algorithm does not preserve the underlying probability distribution of the data, and there is a concern of falsely rejecting the null hypothesis. In an effort to avoid this, the precision of the data has not been limited (the data have not been coarse-grained) as to attempt to keep the number of unique time series values the same for the original and surrogate data [24].  Figure 7 shows typical results for original time series data tested using Algorithm 1 and in this case, the null hypothesis was not rejected. This was true for three subjects in the case of M-L movements and for three in the A-P case. Data for which tests found evidence to reject the null hypothesis are considered questionable at this point, and further examination is required. It is interesting that a portion of the questionable data has non-Gaussian probability distributions (strong fat tails are observed). Overall, the purpose for this hypothesis testing was to mainly check whether the underlying behavior is indicative of a stochastic signal so that our main goal of exploring temporal scaling behavior has proper context.

**Figure 8:**
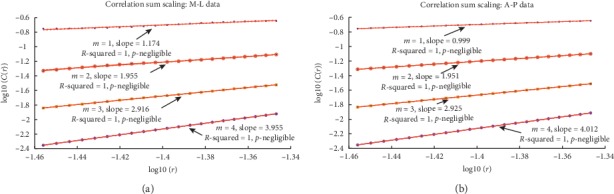
(a, b) Examples of scaling behavior for the correlation sums for M-L and A-P data using the log − log plots. Linear regressions were performed at the 95% level of confidence showing that the correlation sum scale with the embedding dimensions very well in these cases. This is indicative of stochastic behavior underlying these data.

As a final, and somewhat informal, check for stochasticity, we estimated the correlation integral to see if it scales with the embedding dimension for each data set. For this, the correlation sum, *C*(*r*), was used as the estimator. Given *N* data values in some embedding space of dimension, *m*, this sum is given by

(1) $$\begin{array}{c} C\left(r\right)=\frac{2}{N\left(N-1\right)}\overset{N}{\underset{j=1}{\sum }}\overset{N}{\underset{i=j+1}{\sum }}\Theta \left(r-r_{ij}\right), \end{array}$$

where Θ is the Heaviside function and *r*_*ij*_ is the distance between the *i*^th^ and *j*^th^ data points [26]. This distance is given by some norm (we will use the Euclidean norm). With this, *C*(*r*) gives the proportion of pairs of points that fall within the distance, *r*, of one another [45]. As *N*⟶*∞*, it converges to the correlation integral. For a stochastic system, *C*(*r*) ~ *r*^*m*^ for large *N* [50]. As mentioned earlier, calculating *C*(*r*) was done using the usual procedures found in the literature [26, 34, 46, 47].  Figure 8 gives a visual summary of the scaling behavior, for example, M-L and A-P data sets. Scaling indicative of stochastic behavior underlying the data is seen. Regressions performed for the correlation sums of eight other time series (primarily for A-P motion) produced such convincing results. For the other data not being used for this study, the stochastic behavior was not so strongly indicated (more testing needs to be done).

From this point on in our study, we will only be considering the data sets which fall in one of two categories: ones for which surrogate testing found the null hypotheses unable to be rejected for Algorithm 0 or 1—either being consistent in character to IID noise or linearly filtered IID noise. These also have the additional positive check for stochasticity discussed above. This leaves the study with three data sets (one M-L set and two A-P sets) under the criterion for Algorithm 0 and six data sets (three M-L sets and three A-P sets) under the criterion for Algorithm 1.

## 3. Scaling Behavior

### 3.1. A Brief Look at the RMS Jerk

In an effort to gain a general appreciation for movement smoothness and any temporal scaling behavior for our work, we first examined the dimensionless root-mean-square (rms) jerk over a range of time scales. Inspired by the discussion in [30], we defined the RMS jerk for a realization of a discrete set of *N* force values as

(2) $$\begin{array}{c} {\overset{¯}{J}}^{\gamma }\left(\tau \right)=\frac{\langle J^{\gamma }\left(\tau \right)\rangle }{\text{max}\left(\langle J^{\gamma }\left(\tau \right)\rangle \right)},\;\tau =nT_{\text{s}},\\ \langle J^{\gamma }\left(n\right)\rangle ={\left[\frac{1}{N-n}\overset{N-n}{\underset{i=1}{\sum }}J_{i}^{\gamma }{\left(n\right)}^{2}\right]}^{1/2}, \end{array}$$

where

(3) $$\begin{array}{c} J_{i}^{\gamma }\left(n\right)=\frac{f_{i+n}^{\gamma }-f_{i}^{\gamma }}{nT_{\text{s}}}, \end{array}$$

τ is the time scale over which the average is taken, *T*_s_ is the sampling period, *n* is an integer such that *n* < *N*, *γ* indicates M-L or A-P motion, and *f* is the force. Note that our version of the rms jerk is normalized such that its maximum value is 1.00 (max(〈*J*^*γ*^(*τ*)〉) is taken over *τ* for a given realization of a set of force values).

**Figure 9:**
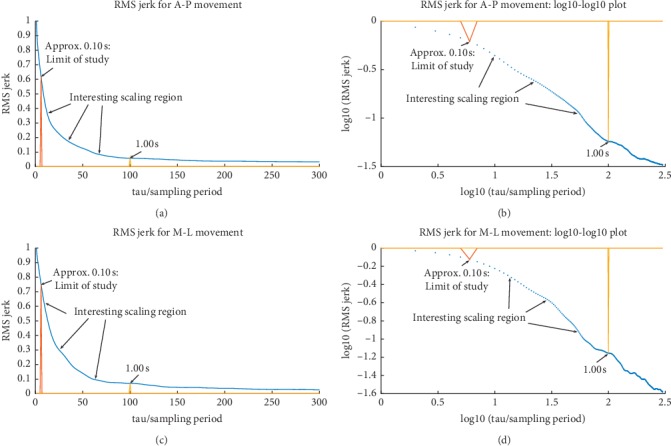
Examples of rms jerk data are plotted as a function of time scale along with the corresponding log-log plot for better visual suggestion of scaling behavior. In general, each depiction is fairly typical of all of the data in terms bulk features concerning shape. A particular range of time is denoted “interesting” in which scaling behavior needs to be explored for each data set. Linear regression of these profiles does not indicate common scaling parameters for such fine-grained features. (a) A-P movement data. (b) log-log plot of values in (a). (c) M-L movement data. (d) log-log plot of values in c.

The results of a visual investigation of the rms jerk are summarized in Figure 9. For data with a bounded set of force differences, the falloff seen in a linear-linear plot of the rms jerk is to be expected as, on average, motion becomes smoother for longer time scales. Certainly, the rms jerk provides a rather coarse view of force variations simply by its construction. Again, we used this approach to provide mainly an overall take on the jerk. In particular, Figure 9 shows two typical cases (linear-linear along with corresponding log-log plots) in which a particular “interesting” span of time common to all data sets is denoted. The lower bound, about 0.1 s, has been chosen as a temporal limit due to machine noise discussed earlier. The upper value of 1.0 s has been taken through inspection to be a time scale at which a transition takes place to a region in which the magnitudes of the jerk and force change relatively slowly. The exact profile of this region does depend on the subject as scaling properties vary; yet, the gross behavior is quite similar. The “interesting” region is so-named as it contains varying rates of change for the magnitudes of the jerk and force. As for the region of time scales beyond 1.0 s, the bulk, but not the fine, structure is similar for all subjects. This is not to say that this region will not be useful to study. Indeed, informative scaling information for this region is discussed below. It is simply that linear regression of each of the log-log plot's “interesting” regions indicates not only more than one scaling exponent but also significantly different exponents for each data set. Scaling behavior regarding temporal correlations over these regions as well as those beyond the 1.0 s scale will be explored in Section 3.2 using detrended fluctuation analysis.

### 3.2. Detrended Fluctuation Analysis

The temporal correlations we are interested in exploring can be seen to be related to its scaling properties referred to above. In particular, physiological signals can display scale invariance [51]. This happens when, for example, a time series structure occurring over some time interval also occurs over intervals of other sizes. So, for a time-dependent signal, *f*(*t*), *f*(*λt*)=*λ*^*α*^*f*(*t*) where *α* is considered a scaling parameter or exponent [28, 52]. We would like to examine such behavior for our data.

In short, detrended fluctuation analysis (DFA) is a technique which is useful for exploring temporal correlations [28, 29]. With this method, the time series with *N* samples is integrated. Then, the resulting series, {*x*(*i*)}, is broken up into a set of non-overlapping windows each consisting of *n* points. A (least squares) linear fit is performed over each window to reveal the local trend, *x*_*n*_(*i*), for each window. The root-mean-square average of the residuals is calculated giving the rms fluctuation:

(4) $$\begin{array}{c} F\left(n\right)=\sqrt{\frac{1}{N}{\overset{N}{\underset{i=1}{\sum }}\left(x\left(i\right)-x_{n}\left(i\right)\right)}^{2}}. \end{array}$$

This calculation is done for all time scales, *n* [47]. How these average fluctuations scale with *n* (*F*(*n*) ~ *n*^*α*^) presents the character of temporal correlations found in the original time series. DFA can be applied to a variety of signals including those with a stochastic fractal nature which may be stationary or not. For example, the scaling may be indicative of fractional Brownian motion (fBm) or fractional Gaussian noise (fGn) [29, 33, 53]. Given our positive tests for stochasticity discussed above, the results of the DFA will be interpreted in this context. For the scaling exponent, *α* (the slope of the log (*F* (*n*)) − log (*n*) plot), the following holds [54, 55].

- *α*=0.5 indicates a signal of white noise or an integrated signal which corresponds to a random walk. The autocorrelation relation is 0 for this signal.
- 0.5 < *α* < 1 corresponds to a positively correlated (power law) or persistent (stationary) fGn signal. This means that increases (decreases) in the signal will, on average, be followed by increases (decreases).
- 0 < *α* < 0.5 corresponds to a negatively correlated (power law) or antipersistent (stationary) fGn signal. So, increases (decreases) in the signal will, on average, be followed by decreases (increases).
- *α*=1 is indicative of pink noise.
- *α* > 1 corresponds to fBm, a nonstationary signal with correlations but not of power law form.
- *α*=1.5 indicates Brownian motion.

**Figure 10:**
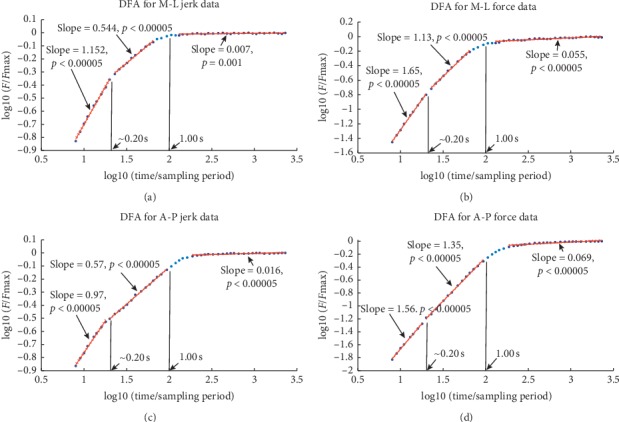
Typical fluctuation profiles generated by performing a detrended fluctuation analysis on our data. Temporal scaling regions common to all data are shown. (a and c) Results for M-L and A-P jerk data, respectively, indicating these realizations are characteristics of (stationary) fractional Gaussian noise. The 95% confidence bounds included on the plot yet are barely visible. Moreover, for the analysis seen in (a), one can argue that a crossover from persistent to antipersistent behavior is made at the time scale of 1.00 s. This is true for other jerk data (Table 1) for both the time scales, 0.20 s and 1.00 s. (b, c) The results of the DFA for the force corresponding to the jerk. These plots correspond to a character of (nonstationary) fractional Brownian motion for the shorter time scales. (a) Example of DFA for M-L jerk data. (b) DFA for M-L force corresponding to the jerk analyzed in (a). (c) Example of DFA for A-P jerk data. (d) DFA for A-P force data corresponding to the jerk analyzed in (c).

**Figure 11:**
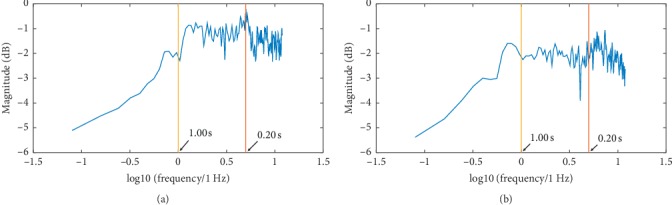
Plots of the power spectral densities typical of our jerk data. A simple visual inspection confirms the existence of transitions between regions of differing temporal correlations particularly for the time scales discussed above. The power spectral density for (a) M-L data and (b) A-P data

Figure 10 offers two typical examples of DFA for our data. DFA profiles for M-L and A-P jerk and associated force data are shown. Three common temporal scaling regions were revealed with 0.160 s being the limit of resolution for the analysis (clearly above the limit imposed by issues with machine noise). The scaling boundary values, 0.20 s and 1.00 s, were found by visual inspection and are, therefore, approximate, yet, certainly fall in or very close to regions of the scaling transition observed in all of the data. The power spectral density (PSD) for jerk data (again, estimated using Welch's method [38]) is presented in Figure 11 to serve as visual confirmation of the existence of the transitions, given the obvious changes in the scaling behavior of the PSD with frequency [23]. The same transition structure can be noted for the DFA concerning the force (Figures 10(b) and 10(d)). Given its nonstationary character, we would like to reserve any more comment other than to say that a scaling transition at the 1.00 s mark is robust among the force data and that the DFA indicates behavior corresponding to fBm for time scales under 1.00 s.

The 1.00 s scale does mark a scaling transition as suggested in our smoothness analysis. The 0.20 s scale revealed itself through DFA. We will label the region for which the time scales are less than 0.20 s Region 1, Region 3 will correspond to scales above 1.00 s, and Region 2 denotes the interval in between the other two. As shown in Table 1, the scaling exponents found for Region 1 for the jerk data are primarily characteristics of persistent fGn for the sets concerning M-L movement, thereby indicating correlated changes in force over time (Table 2, presented in Appendix, lists the F-statistics associated with the linear regressions performed to find the exponents). With exponents also corresponding to fGn, the exponents seen for Region 2 correspond to anticorrelated, (what could be argued to be) uncorrelated, and correlated behaviors. So, one may or may not measure a persistent-to-antipersistent or persistent-to-uncorrelated crossover near the 0.20 s time scale. Also, it is, obviously, difficult to generalize concerning the changes in force on these time scales. Region 3 has relatively low value exponents indicative of anticorrelated fGn. Perhaps, such low exponent values of the third region suggest some periodic trend as studied in [56] in which a flat DFA profile was seen to be indicative of a sinusoidal trend. However, this would violate the time series' stationary character which was successfully tested for. Still, possibly quasiperiodic behavior exists as a low exponent value is indicative of a slowly varying accumulation of fluctuations which could be found with a quasirecurrent signal structure. This would, then, correspond to a quasioscillatory force.

## 4. Discussion

Overall, for a sizable subset of our total data set, we have found behavior strongly suggestive of fGn for the jerk associated with human one-leg stance. That is, the jerk data under study have been found to be stationary and stochastic with persistent and antipersistent behavior. In the least, these findings suggest, as Collins and De Luca concluded (for bipedal quiet stance) that the system of postural control could be modeled as a stochastic process [27, 57]. Also, the quality of stationarity implies that (after transients) the jerk is constant in variance. This is interesting as the unipedal balance study presented in [22] mentioned in our introduction section recognized a long-term “static phase” for all of their subjects during which force variability remained at a certain level. Our results suggest that the force increments display such behavior, whereas the force exhibits nonstationarity (variance can be time-dependent). This can be seen to make sense for the generally steady balance sessions we observed if steadiness is defined in terms of the jerk and its variability.

Moreover, three temporal scaling regions common to our data have been identified (Table 1). For both A-P and M-L movements, the fastest time scale corresponds to behavior with persistent correlations while the slowest corresponds to antipersistent motion. Between these time scales, we find behavior indicative of persistent, antipersistent, and (suggestions of) uncorrelated behaviors with no obvious patterns concerning A-P and M-L motions (no obvious tendency for one motion to favor one type of correlation). Given earlier studies of postural stance, it is not surprising to find such scaling behavior. Duarte and Zatsiorsky[58] found long-range correlations over various time scales in the case of bipedal stance, however over time scales much longer than those considered for our work. In our case, given the small size of the fastest scale, one may claim that this is a region of autonomic activity, whereas for the longest scales, voluntary muscle behavior is in play [59]. So, it may be that fast persistent jerks, or changes in force, occur along with relatively slow antipersistent jerks possibly indicative of a quasiperiodic “to and fro” motion alluded to earlier in this paper (we did attenuate certain frequencies and narrow frequency bands in the time series and found no significant resulting change in the DFA that would clearly indicate an outstanding periodic oscillation was present; indeed, such an oscillation should lead to a detectable violation of the stationarity condition [26]). Also, the temporal scale bridging the fast and slow scale extremes is interesting. This region's scaling character, with cases of persistence, antipersistence, and uncorrelated behaviors (Table 1), is not consistent among the data and is certainly in need of better understanding. This may follow a better overall understanding of the underlying mechanism which drives the data.

In addition, any crossover between persistent and antipersistent behaviors arouses curiosity. In a study about human quiet standing by Delignieres et al. [4] in which the work of Collins and de Luca [2] is revisited, the crossover behavior seen regarding the differenced time series of COP data implied a velocity-based control strategy being used for postural sway. In this spirit, our differenced time series of force data (which, again, are directly proportional to the jerk) may play a role similar to that of the differenced COP data of the study just mentioned. A transition from persistence on one set of time scales to antipersistence on longer time intervals implies that the data are bounded [60]. So, for our data for one-leg stance, one could see the jerk evolving between upper and lower limits which correspond to a control on the changes in force over time. On one set of time scales, force increments are positively correlated, whereas on longer time intervals, force increments become anticorrelated. This idea of a jerk-based control needs further study and will be pursued in the near future.

It is also interesting to question whether the jerk is related to *trembling*; movements away from a reference position about which the body would attempt to maintain equilibrium [61]. Zatsiorsky and Duarte found evidence supporting the idea that trembling along with the motion of the reference point corresponds to reasons why the body sways during bipedal quiet stance. They also found trembling to be a “high frequency process” in light of other relevant time scales and correlates with the horizontal ground reaction force. Trembling has been quantified as a deviation from an instantaneous equilibrium point determined during A-P sway [61]. Although the work of these authors corresponds to bipedal quiet stance, we find that a brief comparison with their results to be interesting. Their power spectral density for average trembling indicates activity mainly between 0 and 2.0 Hz (with a peak at approximately 0.40 Hz). This range overlaps Regions 2 and 3 of our study for A-P motion encompassing the transition from higher to lower DFA exponents with the larger exponents close in scaling to that of white noise to negatively correlated noise. In fact, the region corresponding to negatively correlated noise may correspond to a quasiperiodic oscillation as mentioned above (the autocorrelation function, such as in Figure 4(d), may suggest this behavior with marginal quasiperiodic long-range correlations—more investigation is needed). Any wavering, or “pendulum-like” motion, associated with trembling may be related to the jerk on these time scales [61].

Overall, efforts such as more extensive surrogate data testing are needed (in particular, for nonstationary data) with the goal of being informed as to the specific nature of this mechanism rather than only the general character (stochasticity and stationarity) which satisfied the requirements of this study. Additionally, a simple (autoregressive) model of the behavior we have observed needs to be developed to test ideas concerning that underlying mechanism. These efforts are currently in progress.

## Figures and Tables

**Table 1:** The results of the detrended fluctuation analysis.

Dataset		Region 1		Region 2		Region 3	
No.	Movement	Exponent	*R* ^2^	Exponent	*R* ^2^	Exponent	*R* ^2^
1	A-P	0.97	0.995	0.57	0.998	0.016	0.700
2	A-P	0.76	0.991	0.56	0.996	0.034	0.820
3	A-P	0.90	0.998	0.42	0.998	0.016	0.519
4	A-P	0.95	0.996	0.63	0.997	0.039	0.698
5	A-P	0.63	0.998	0.40	0.993	0.050	0.627
6	M-L	0.80	0.991	0.35	0.988	0.029	0.775
7	M-L	0.94	0.992	0.77	0.993	0.042	0.410
8	M-L	0.95	0.996	0.78	0.988	0.025	0.751
9	M-L	1.15	0.996	0.544	0.995	0.007	0.363

Scaling exponents are shown for each of the three scaling regions. These exponents were found using linear regression to 95% confidence limits. For each fit, the *p* value <0.05 (the F statistics are presented in Appendix). The relevant fit statistics are listed. Also, notice that crossovers from persistence to antipersistence can be found between various regions. Region 1: 0.10 s < *t* < 0.20 s; Region 2: 0.20 s < *t* < 1.00 s; Region 3: *t* > 1.00 s.

**Table 2:** The *F*-statistics associated with the regressions used in the detrended fluctuation analysis.

Dataset		Region 1	Region 2	Region 3
#	Movement	*F*-statistic	*F*-statistic	*F*-statistic
1	A-P	*F* (2, 9) = 1290	*F* (2, 14) = 5540	*F* (2, 23) = 49.0
2	A-P	*F* (2, 7) = 530	*F* (2, 12) = 2550	*F* (2, 14) = 54.7
3	A-P	*F* (2, 6) = 2220	*F* (2, 13) = 5200	*F* (2, 14) = 13.0
4	A-P	*F* (2, 6) = 1030	*F* (2, 13) = 3590	*F* (2, 14) = 27.7
5	A-P	*F* (2, 7) = 423	*F* (2, 12) = 1500	*F* (2, 14) = 22.9
6	M-L	*F* (2, 8) = 641	*F* (2, 12) = 837	*F* (2, 18) = 55.0
7	M-L	*F* (2, 8) = 758	*F* (2, 9) = 956	*F* (2, 18) = 11.1
8	M-L	*F* (2, 8) = 1590	*F* (2, 9) = 558	*F* (2, 15) = 48.3
9	M-L	*F* (2, 9) = 1750	*F* (2, 10) = 1590	*F* (2, 26) = 13.7

Region 1: 0.10 s < *t* < 0.20 s; Region 2: 0.20 s < *t* < 1.00 s; Region 3: *t* > 1.00 s.

## Data Availability

The jerk and supporting force data (in *∗*.xlsx format) used to support the findings of this study have been deposited in the Computational and Mathematical Methods in Medicine repository found at https://www.dropbox.com/sh/31yo554lkzf32yp/AAC98A4I9jlBdDCkmVHP1ePHa?dl=0. These are publicly available files. The data associated with this study can also be accessed by contacting the corresponding author at matthew.semak@unco.edu.
